# Identification of candidate inverse miRNA–mRNA pairs and an internally validated tissue-based diagnostic model for benign and malignant breast lesions

**DOI:** 10.3389/fmolb.2026.1952773

**Published:** 2026-10-23

**Authors:** Xiaoyan Zhao, Hui Feng, Xingxing Wu, Dan Zhao

**Affiliations:** 1 Department of Breast Surgery, Shanxi Provincial Children’s Hospital, Shanxi Provincial Maternity and Child Healthcare Hospital, Taiyuan, Shanxi, China; 2 Department of Breast Surgery, Taiyuan Maternal and Child Health Hospital, Taiyuan, Shanxi, China

**Keywords:** benign breast lesion, breast cancer, candidate inverse miRNA–mRNA pair, diagnostic model, Lasso logistic regression, miRNA, mRNA, RT-qPCR

## Abstract

**Background:**

Differentiating malignant breast tumors from benign breast lesions remains clinically important when imaging or pathology is indeterminate. We evaluated candidate inverse miRNA–mRNA expression pairs as a tissue-based adjunctive molecular approach intended to complement, not replace, histopathological diagnosis, with particular relevance to diagnostically challenging lesions such as BI-RADS four or radiologic–pathologic discordant cases.

**Methods:**

Public microRNA (miRNA) and messenger RNA (mRNA) datasets were used for candidate discovery and prioritization. An independent reverse-transcription quantitative polymerase chain reaction (RT-qPCR) tissue cohort included 150 breast-lesion samples (68 benign and 82 malignant). Ten candidate axes were measured. A least absolute shrinkage and selection operator (LASSO) logistic-regression model was developed in the training cohort and tested without marker reselection, coefficient refitting, or threshold re-optimization in the internal-validation cohort. Public datasets were used primarily for tumor-versus-normal discovery, whereas benign-versus-malignant evaluation was performed in the independent tissue cohort.

**Results:**

miR-205-5p/HMGB3 and miR-96-5p/FOXO1 showed the strongest inverse-expression candidate evidence. The final model retained miR-205-5p, HMGB3, miR-96-5p, FOXO1, and miR-21-5p. In internal validation, the area under the receiver operating characteristic curve (AUC) was 0.894 (95% confidence interval 0.788–0.975), with accuracy 0.844, sensitivity 0.840, and specificity 0.850.

**Conclusion:**

A five-marker tissue model discriminated benign from malignant breast lesions in internal validation. miR-205-5p/HMGB3 and miR-96-5p/FOXO1 were the leading inverse-expression candidates. External validation and functional confirmation are required before clinical use.

## 1 Introduction

Breast cancer remains one of the most frequently diagnosed cancers worldwide and continues to create a substantial diagnostic burden (Bray et al., 2024). In routine practice, the Breast Imaging Reporting and Data System (BI-RADS) standardizes imaging-based risk assessment, but suspicious or indeterminate lesions still require tissue sampling and pathological confirmation (D'Orsi et al., 2013). A tissue-based molecular model should therefore complement, not replace, histopathology, particularly in diagnostically difficult lesions. Benign breast lesions are also heterogeneous, ranging from non-proliferative changes to proliferative lesions or atypia with different long-term risks (Hartmann et al., 2005).

MicroRNAs (miRNAs) are short non-coding RNAs that mainly regulate gene expression through post-transcriptional effects on messenger RNAs (mRNAs). Their dysregulation has been widely reported in breast cancer and has been associated with tumor subtype, receptor status, proliferation, invasion, and treatment-related behavior (Calin and Croce, 2006; Iorio et al., 2005; Dvinge et al., 2013; Lowery et al., 2009). Because one miRNA can influence multiple targets, paired miRNA–mRNA evaluation may provide more interpretable biomarker information than isolated markers (Ahmad et al., 2022; Zaidi et al., 2023).

Candidate inverse miRNA–mRNA pairs provide an interpretable biomarker structure when differential expression, target-resource support, and inverse expression are considered together. However, negative expression correlation alone cannot establish direct miRNA-mediated repression, particularly when correlations are calculated across diagnostically distinct groups. Integrated miRNA–mRNA analysis is therefore best used to prioritize candidate axes for laboratory and clinical validation rather than to establish mechanism by itself (Huang et al., 2022; Stingo et al., 2010).

Previous breast cancer studies have shown that miRNA signatures can distinguish tumor subgroups and may support diagnostic classification (Adhami et al., 2018). Negatively associated miRNA–mRNA pairs have also been investigated in the distinction between breast cancer and benign breast disease (Li et al., 2023). Nevertheless, discovery evidence, tissue-level expression, diagnostic modeling, and mechanistic regulation represent different levels of evidence. The present study therefore uses the term candidate inverse miRNA–mRNA pair unless direct functional evidence is available. Recent precision-oncology reviews likewise emphasize both the diagnostic promise of compact miRNA signatures and the need for transparent feature selection, calibration, and independent validation (Zehra et al., 2026).

The present study was designed to identify candidate inverse miRNA–mRNA pairs associated with malignant breast tumors and to develop an internally validated tissue-based molecular model for distinguishing malignant tumors from benign breast lesions. Public expression datasets were used for tumor-associated discovery and target-supported prioritization, whereas the independent RT-qPCR cohort provided benign–malignant tissue evaluation and model development. The intended clinical position is an adjunctive tissue-based model for diagnostically challenging lesions rather than a replacement for imaging or histopathology (Shafeeque et al., 2025; Shafeeque et al., 2026).

## 2 Methods

### 2.1 Study design and ethical approval

This diagnostic biomarker and prediction-model study was designed to identify candidate inverse miRNA–mRNA pairs associated with malignant transformation in breast lesions and to develop a tissue-based molecular model for distinguishing benign from malignant lesions. Public transcriptomic datasets were used to define tumor-associated miRNA and mRNA expression direction, target-supported interactions, and tumor-level inverse-correlation patterns. An independent RT-qPCR tissue cohort from Shanxi Provincial Children’s Hospital was then used for benign–malignant expression assessment and internal model development. Histopathology remained the diagnostic reference standard; the molecular model was intended only as an adjunctive tissue-based tool.

The final histopathological diagnosis from surgical excision or core-needle biopsy was the reference standard. The study was approved by the ethics committee of Shanxi Provincial Children’s Hospital (Approval No. SPCH2025068HBSDC). Reporting followed diagnostic-accuracy and multivariable prediction-model principles (Bossuyt et al., 2015; Collins et al., 2015). The tissue-validation cohort covered 6 January to 31 October 2025, and the database was locked on 15 November 2025 before statistical analysis.

### 2.2 Public transcriptomic datasets and differential-expression analysis

Public breast tumor, adjacent normal breast tissue, apparently normal breast tissue, and benign/non-cancer breast-lesion expression datasets were retrieved from the Gene Expression Omnibus (GEO). The discovery layer included miRNA datasets GSE45666, GSE26659, and GSE58210, the benign–malignant direction-check dataset GSE144534, and mRNA datasets GSE109169 and GSE139038. The Cancer Genome Atlas Breast Invasive Carcinoma dataset (TCGA-BRCA) was used only for paired tumor-level miRNA–mRNA correlation and was not treated as a benign comparator. Dataset sources, platforms, sample types, and analytic roles are detailed in Supplementary Table S1. Pair-specific TCGA complete-case counts were not available in the retained source files and are therefore not reported.

GEO series matrix files and platform annotation files were processed in R 4.4.2 using GEOquery 2.74.0 and Biobase 2.66.0. miRNA probes were mapped to official mature miRNA names, and mRNA probes were mapped to HGNC-approved gene symbols. When multiple probes mapped to the same miRNA or gene, the probe with the highest median absolute deviation across samples was retained to preserve the most informative expression signal. Expression matrices were log2 transformed when required and quantile-normalized within each platform using preprocessCore 1.68.0. Cross-platform expression matrices were not pooled for primary differential-expression testing.

Samples were removed before analysis if more than 20% of expression values were missing, if diagnostic or tissue annotation was unclear, or if duplicated sample identifiers were present. miRNAs and mRNAs detected in fewer than 20% of samples within a dataset were also removed. Differential-expression analysis was performed separately for each dataset using limma 3.62.2 (Ritchie et al., 2015). Benjamini–Hochberg correction was applied within each dataset. miRNAs and mRNAs were retained for cross-dataset screening when the adjusted *P* value was <0.05, the absolute log2 fold change was ≥0.40, and the disease-associated direction was consistent in at least two relevant discovery datasets. GSE144534 was used as a secondary benign–malignant tissue-level direction check for candidate miRNAs rather than as the only discovery source. ComBat adjustment in sva 3.54.0 was used only for exploratory cross-platform visualization after within-platform normalization and was not used to generate the primary differential-expression statistics.

### 2.3 miRNA–mRNA pair filtering, network analysis, and pathway annotation

Candidate inverse miRNA–mRNA pairs were generated by intersecting three evidence layers: differentially expressed miRNAs, differentially expressed mRNAs, and target-interaction support. Experimentally supported interactions in miRTarBase 2025 were prioritized (Cui et al., 2025). Predicted or sequence-based interactions were checked using TargetScanHuman 8.0 and miRDB 6.0, and Ago/CLIP-supported interactions were checked using ENCORI/starBase 3.0 (Agarwal et al., 2015; Chen and Wang, 2020; Van Nostrand et al., 2020). A predicted pair without experimental support was retained only when supported by at least two prediction or CLIP-based resources.

The main screen was restricted to inverse disease-associated direction. Upregulated miRNAs were paired with downregulated mRNAs, and downregulated miRNAs with upregulated mRNAs. Pearson correlation was then calculated in paired-expression data as a prioritization criterion. A pair was retained when Pearson r was <−0.20, *P* was <0.05, and at least 80% of paired samples had both values available. Because pooled correlation can partly reflect diagnostic-group separation, these coefficients are treated as descriptive inverse-expression evidence rather than proof of direct regulation. Accordingly, pair nomenclature throughout the manuscript was restricted to candidate inverse-expression pairs unless functional evidence was available.

Candidate axes were prioritized using inverse expression direction, negative-correlation strength, target-database support, repeated discovery-dataset support, degree centrality, and betweenness centrality. Axes selected for RT-qPCR evaluation also had to show a biologically interpretable malignant-associated direction and target-interaction support. Supplementary Table S2 provides the relative ranking of the ten measured RT-qPCR panel axes.

Pathway annotation of candidate mRNA targets was performed with clusterProfiler 4.14.4 and org.Hs.e.g.,.db 3.20.0 in R 4.4.2 (Yu et al., 2012). Gene Ontology (GO) biological-process and Kyoto Encyclopedia of Genes and Genomes (KEGG) enrichment analyses used Benjamini–Hochberg adjusted *P* < 0.05. Enrichment was interpreted as functional context for candidate prioritization rather than evidence of direct miRNA-mediated regulation.

### 2.4 Tissue-validation cohort and clinical variables

The tissue-validation cohort comprised 150 breast-lesion specimens obtained at the Department of Breast Surgery, Shanxi Provincial Children’s Hospital (Shanxi Provincial Maternity and Child Healthcare Hospital): 68 benign lesions and 82 malignant tumors. Index tissue was obtained either by core-needle biopsy or during surgical excision, and patients who had received neoadjuvant systemic therapy before tissue sampling were excluded. Thus, the cohort included diagnostic biopsy and surgical-excision material rather than a single postoperative specimen class. The retained study database did not consistently encode fresh/frozen versus paraffin-embedded status for every specimen; preservation format was therefore not used as an analytical variable. Samples were then assigned by diagnosis-stratified randomization to a training cohort (n = 105) and an internal-validation cohort (n = 45).

Eligible patients were female patients with an available index breast-lesion tissue specimen, a complete final histopathological diagnosis, and sufficient RNA for RT-qPCR analysis. Benign lesions included fibroadenoma, fibrocystic change, adenosis, intraductal papilloma without malignant transformation, and benign phyllodes tumor. Malignant tumors included invasive ductal carcinoma, invasive lobular carcinoma, ductal carcinoma *in situ*, and other pathologically confirmed breast carcinomas. The final pathology report from surgical excision or core-needle biopsy defined the diagnostic group.

Patients were excluded for recurrent breast cancer, distant metastatic disease at first presentation, neoadjuvant systemic therapy before tissue sampling, active mastitis or abscess involving the sampled lesion, ambiguous pathological classification, insufficient tissue for RNA extraction, RNA integrity number <5.5, estimated target-lesion cellularity <30%, missing expression for any prespecified core-axis marker, or missingness in more than 20% of measured molecular variables. When multiple lesions were present, the sampled lesion corresponding to the final pathological diagnosis was used as the index lesion. If more than one lesion was sampled during the same diagnostic episode, one lesion was retained according to the highest BI-RADS category and then the largest diameter.

Clinical variables included age, menopausal status, BI-RADS category, lesion size, histological subtype, and target-lesion cellularity. For malignant tumors, estrogen receptor (ER), progesterone receptor (PR), human epidermal growth factor receptor 2 (HER2), and Ki-67 were extracted from routine immunohistochemistry reports. ER and PR positivity were defined as nuclear staining in ≥1% of tumor cells (Allison et al., 2020), and HER2 status followed immunohistochemistry and *in situ* hybridization results when available (Wolff et al., 2023). Variable definitions, coding rules, quality-control thresholds, and analytic roles are provided in Supplementary Table S3.

### 2.5 RNA extraction, RT-qPCR measurement, and assay quality control

Breast-lesion tissue designated for molecular analysis was macrodissected before ribonucleic acid (RNA) extraction, and hematoxylin-and-eosin sections were reviewed to confirm target-lesion content. Small-RNA-containing total RNA was extracted using the mirVana miRNA Isolation Kit (Invitrogen/Thermo Fisher Scientific; AM1560). RNA concentration was measured with the Qubit RNA HS Assay Kit, purity with the NanoDrop 2000 spectrophotometer, and integrity with the Agilent 2100 Bioanalyzer. The RNA integrity number (RIN) had to be ≥ 5.5; A260/A280, 1.80–2.10; A260/A230, ≥1.70; and total RNA input, ≥100 ng. Additional assay and quality-control information is provided in Supplementary Table S4.

RT-qPCR measured predefined candidate miRNAs and paired mRNA targets in the same tissue specimen. The ten candidate axes were miR-205-5p/HMGB3, miR-96-5p/FOXO1, miR-21-5p/PIK3R1, miR-182-5p/ARRDC3, miR-200c-3p/FBLN5, miR-342-3p/TACC1, miR-195-5p/RACGAP1, miR-139-5p/TPD52, miR-125b-5p/PARP1, and miR-145-5p/TPM3. The prespecified core axes were miR-205-5p/HMGB3, miR-96-5p/FOXO1, and miR-21-5p/PIK3R1. The remaining axes were measured as network-supported or exploratory candidate axes and were eligible for model screening according to the rules described in Section 2.6.

For miRNAs, reverse transcription used the TaqMan Advanced miRNA complementary DNA (cDNA) Synthesis Kit, followed by target-specific TaqMan Advanced miRNA Assays and TaqMan Fast Advanced Master Mix. For mRNAs, reverse transcription used the High-Capacity cDNA Reverse Transcription Kit, and qPCR used PowerUp SYBR Green Master Mix. Amplification was performed on a QuantStudio 5 Real-Time PCR System, with each target measured in technical triplicate. Target-specific identifiers, reference genes, accepted cycle-threshold (Ct) ranges, and repeat rules are listed in Supplementary Table S4. Reporting followed the Minimum Information for Publication of Quantitative Real-Time PCR Experiments (MIQE) recommendations (Bustin et al., 2009).

A qPCR result was accepted when the triplicate Ct standard deviation was ≤0.35. A target was repeated once when the triplicate Ct standard deviation was >0.35. If the repeat assay remained unstable, the target value was recorded as missing. Ct values > 35, undetermined Ct values, and no-template-control amplification before Ct 38 were treated as failed reactions. Inter-plate calibrator drift had to remain within 0.50^−ΔΔCT^; plates exceeding this threshold were repeated. miRNA expression was normalized to the geometric mean Ct of hsa-miR-16-5p and hsa-miR-191-5p. mRNA expression was normalized to the geometric mean Ct of GAPDH and ACTB. Normalized expression was calculated as −ΔCt, so higher values represented higher relative expression.

### 2.6 Diagnostic-model development and validation

The diagnostic model was developed in the training cohort and tested without modification in the internal-validation cohort. Candidate molecular predictors were the RT-qPCR measurements from the ten predefined axes listed in Section 2.5. Candidate-pair evidence and diagnostic-model development were treated as separate analyses: inverse expression and correlation were used for candidate interpretation, whereas model development evaluated which measured markers improved benign–malignant classification. The final score itself used only markers with complete expression values and did not impute any retained molecular predictor.

Molecular markers were standardized with training-cohort means and standard deviations, and the same transformation was applied to the internal-validation cohort. The five markers retained in the final score had complete measurements and were not imputed. Missing clinical covariates (<20%) were imputed only for sensitivity analyses with mice 3.16.0. The primary five-marker model was fitted in the full training cohort (n = 105). Additional full-panel complete-case screening and stability analyses are reported separately in Supplementary Tables S5–S6 and are not used to replace the primary model estimates.

Penalized logistic regression was used for marker selection because several miRNA and mRNA predictors were correlated. LASSO logistic regression was fitted using glmnet 4.1–8, with ten-fold cross-validation repeated 50 times in the training cohort (Friedman et al., 2010). The tuning parameter was selected using the one-standard-error rule to favor a smaller and more stable marker panel. Markers with non-zero LASSO coefficients were then refitted in a conventional logistic-regression model in the training cohort. The diagnostic probability was calculated as 1/(1 + exp[−linear predictor]), where the linear predictor was the logistic-regression intercept plus the weighted standardized marker values.

The malignant-classification threshold was selected in the training cohort using the Youden index and fixed at 0.52 before internal validation. Receiver-operating-characteristic analysis was performed using pROC 1.18.5. AUC 95% confidence intervals were estimated using DeLong’s method (DeLong et al., 1988; Robin et al., 2011). At the locked threshold, accuracy, sensitivity, specificity, positive predictive value (PPV), negative predictive value (NPV), F1 score, and confusion-matrix counts were reported. Wilson 95% confidence intervals were calculated for binomial classification measures. No marker reselection, coefficient refitting, threshold re-optimization, or normalization recalibration was performed in the internal-validation cohort.

Calibration was assessed with the calibration intercept, calibration slope, Brier score, and bootstrap-corrected plots. Decision-curve analysis used threshold probabilities of 0.10–0.90 (Vickers and Elkin, 2006). Prespecified sensitivity analyses adjusted for age and BI-RADS, excluded ductal carcinoma *in situ*, restricted analysis to RIN≥6.0 and target-lesion cellularity≥40%, and repeated the diagnosis-stratified train-validation split 100 times.

All statistical analyses were performed in R 4.4.2. Data management used tidyverse 2.0.0 and readxl 1.4.3, and cohort splitting/model training used caret 6.0–94. Two-sided *P* values <0.05 were considered statistically significant for clinical and model-level analyses. BI-RADS distributions were evaluated with one overall chi-square test rather than separate tests for each category. For the ten candidate-pair correlation tests, Benjamini–Hochberg adjustment was applied as one family of tests; because four raw values were reported only as *P* < 0.001, 0.001 was used conservatively for adjustment and the corresponding adjusted values are reported as upper bounds. Transcriptome-wide screening, target enrichment, and pathway analyses also used Benjamini–Hochberg adjusted *P* values <0.05. All random procedures used fixed random seeds recorded in the analysis script.

Additional analyses included benign- and malignant-group Pearson correlations, pooled partial correlations adjusted for diagnosis, age, and target-lesion cellularity with Benjamini–Hochberg correction; repeated LASSO cross-validation; diagnosis-stratified train-validation repetitions; comparison of BI-RADS-only, clinical, molecular, and combined models; a BI-RADS four subgroup analysis; bootstrap uncertainty for calibration measures; and reporting of five-marker standardization parameters. These supplementary analyses are analytically separate from the prespecified primary performance estimates reported in Sections 3.4–3.5.

## 3 Results

### 3.1 Public expression screening and candidate inverse-pair prioritization

After dataset-level preprocessing, the public discovery layer was used to define cancer-associated expression direction, secondary benign–malignant miRNA direction support, and paired tumor-level miRNA–mRNA correlation. Recurrent differential-expression direction was identified for 43 miRNAs and 612 mRNAs across the discovery datasets. Intersection with miRTarBase, TargetScanHuman, miRDB, and ENCORI/starBase produced 196 target-supported miRNA–mRNA pairs. Of these, 78 showed inverse disease-associated expression direction and 36 met the paired-expression negative-correlation rule. Ten candidate inverse miRNA–mRNA axes were selected for RT-qPCR evaluation according to inverse direction, target-resource support, correlation strength, network position, and biological interpretability. These public-data correlations were used for candidate prioritization and not as evidence of direct regulation. Thus, tumor-versus-normal public discovery and benign-versus-malignant tissue evaluation were treated as distinct analytical stages rather than as interchangeable evidence streams. The overall study workflow and principal data-reduction steps are shown in Figure 1.

**FIGURE 1:**
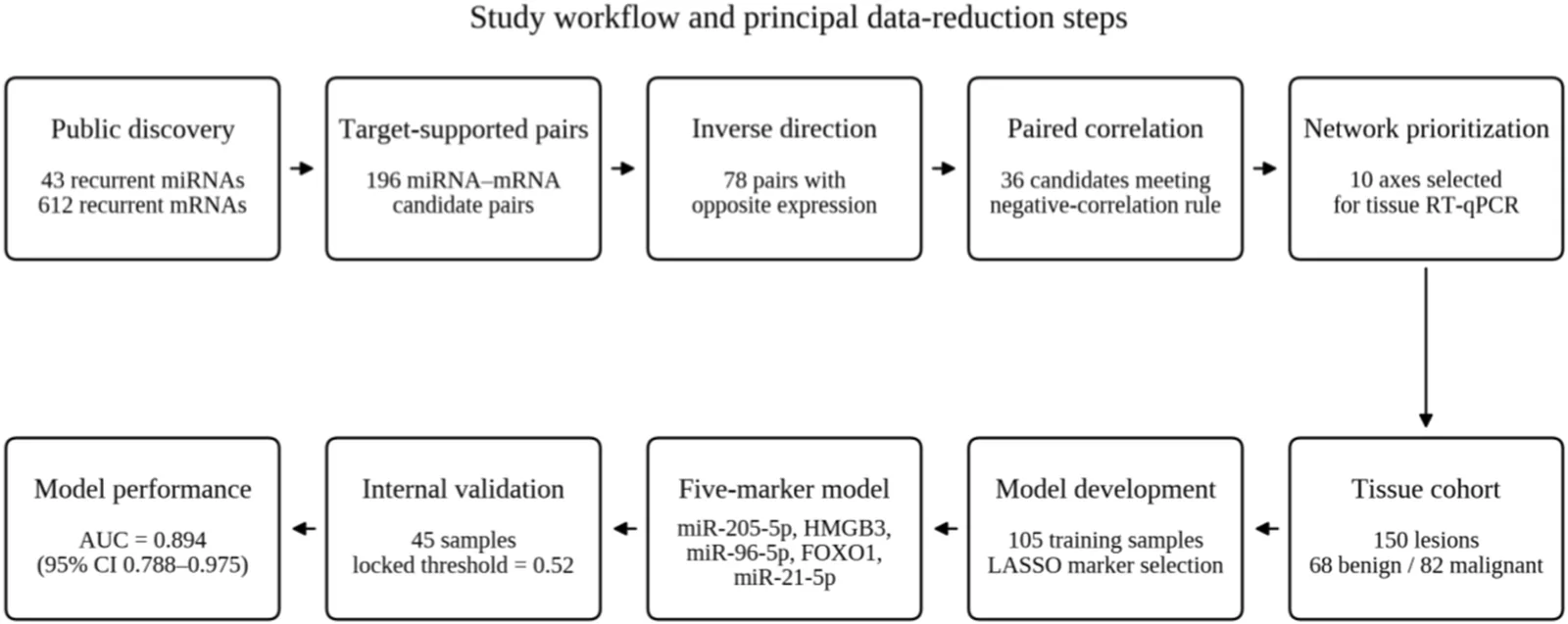
Study workflow and principal data-reduction steps. Abbreviations: miRNA, microRNA; mRNA, messenger RNA; RT-qPCR, reverse-transcription quantitative polymerase chain reaction; LASSO, least absolute shrinkage and selection operator; AUC, area under the receiver operating characteristic curve; CI, confidence interval.

Pathway annotation of candidate mRNA targets supported enrichment in malignant-transformation-related processes, including regulation of cell proliferation, apoptotic signaling, extracellular-matrix organization, epithelial–mesenchymal transition, PI3K/AKT signaling, focal adhesion, and breast cancer-related pathways. The public screening and enrichment results are summarized in Figure 2.

**FIGURE 2:**
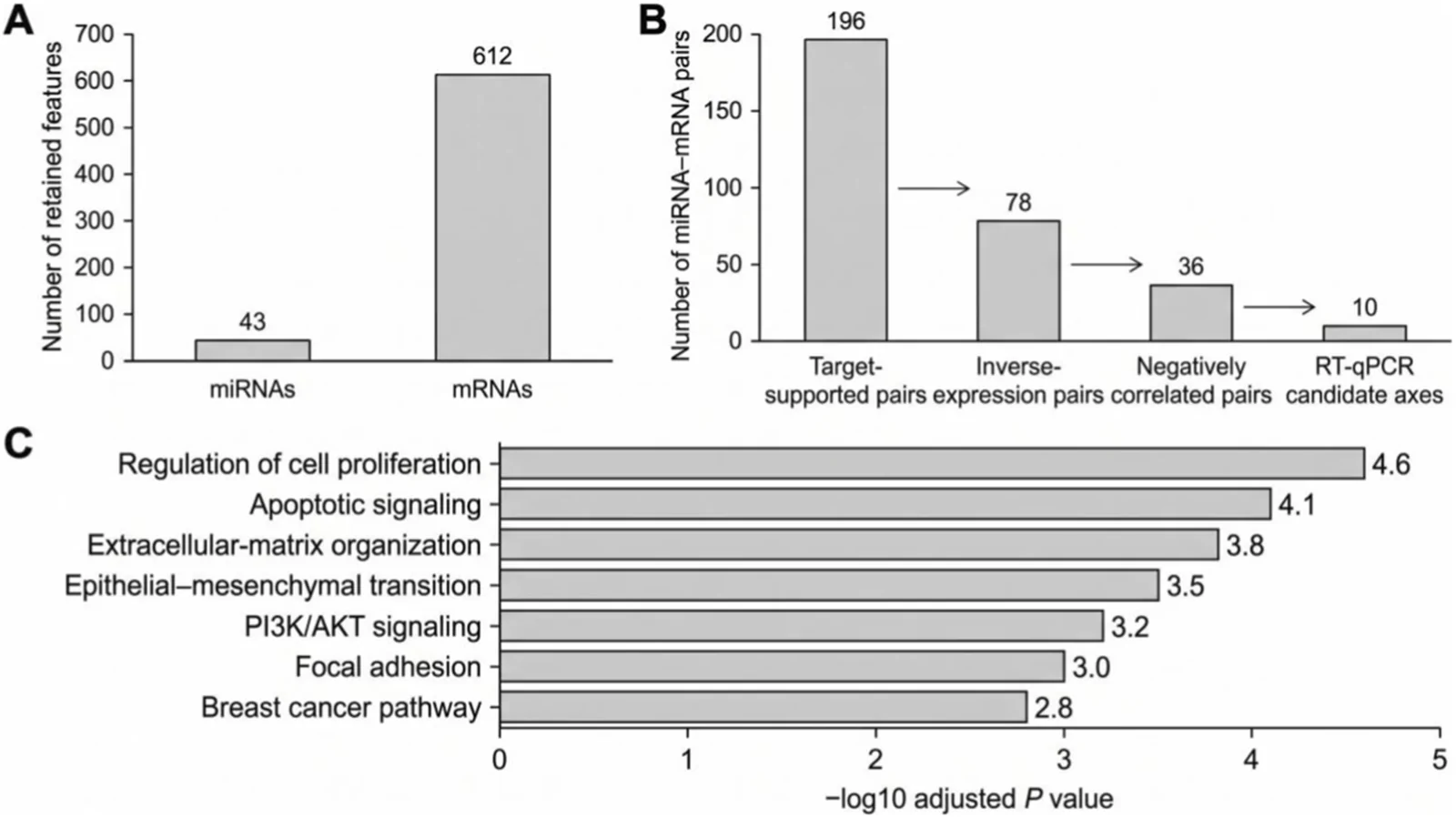
Public discovery, filtering, and enrichment statistics. **(A)** Numbers of retained miRNA and mRNA features after preprocessing and recurrent differential-expression screening. **(B)** Sequential reduction from target-supported pairs to inverse-expression pairs and negatively correlated pairs. **(C)** Top enriched Gene Ontology biological-process and KEGG pathway terms among candidate mRNA targets. Abbreviations: miRNA, microRNA; mRNA, messenger RNA; RT-qPCR, reverse-transcription quantitative polymerase chain reaction; GO, Gene Ontology; KEGG, Kyoto Encyclopedia of Genes and Genomes; PI3K, phosphoinositide 3-kinase; AKT, protein kinase B.

The workflow reduced 196 target-supported pairs to 78 inverse-direction pairs and 36 negatively correlated candidates. Ten axes were carried forward to RT-qPCR evaluation in 150 lesions, and LASSO retained five markers for the final internally validated model.

### 3.2 Tissue-validation cohort and assay quality

The tissue-validation cohort included 150 breast-lesion samples, comprising 68 benign lesions and 82 malignant tumors. Diagnosis-stratified randomization assigned 105 samples to training and 45 to internal validation. Compared with benign lesions, malignant tumors occurred in older patients, had larger lesions, a higher prevalence of postmenopausal status, and higher target-lesion cellularity. BI-RADS four lesions comprised 70 of 150 cases (28 benign and 42 malignant). RNA integrity number did not differ significantly between diagnostic groups. One overall chi-square test confirmed a marked difference in BI-RADS distribution (χ^2^ = 67.54, df = 2, *P* < 0.001).

Benign lesions included fibroadenoma, fibrocystic change, adenosis, intraductal papilloma without malignant transformation, and benign phyllodes tumor. The benign-lesion distribution was fibroadenoma in 31 cases, fibrocystic change in 18 cases, adenosis in 9 cases, intraductal papilloma in 7 cases, and benign phyllodes tumor in 3 cases. Malignant tumors included invasive ductal carcinoma in 60 cases, ductal carcinoma *in situ* in 12 cases, invasive lobular carcinoma in 6 cases, and mucinous carcinoma in 4 cases. Among malignant tumors, ER positivity was recorded in 55 of 82 cases, PR positivity in 49 of 82 cases, and HER2 positivity in 18 of 82 cases. The Ki-67 index was 30.9% ± 17.1%.

No sample was excluded for missing expression of any prespecified core-axis marker. Thirty-eight samples had at least one missing non-core marker, and a complete molecular profile was available in 112 of 150 samples. Importantly, all five markers retained in the final diagnostic score were core-axis markers with complete measurements; no imputation was used when calculating the final score. Cohort characteristics and molecular-quality indicators are shown in Table 1 and Figure 3.

**TABLE 1:** Cohort characteristics and molecular-quality indicators.

Characteristic	Benign lesions n=68	Malignant tumors n=82	*P* value
Age, years	43.2 ± 8.8	54.0 ± 12.1	<0.001
Postmenopausal status, n (%)	18 (26.5)	43 (52.4)	0.002
Lesion size, mm	20.5 ± 7.5	32.4 ± 13.3	<0.001
BI-RADS 3, n (%)	36 (52.9)	0 (0.0)	Overall <0.001
BI-RADS 4, n (%)	28 (41.2)	42 (51.2)	
BI-RADS 5, n (%)	4 (5.9)	40 (48.8)	
RNA integrity number	7.66 ± 0.62	7.52 ± 0.65	0.192
Target-lesion cellularity, %	45.0 ± 13.9	66.0 ± 13.9	<0.001
Complete molecular profile, n (%)	51 (75.0)	61 (74.4)	1.000
Non-core marker missingness, n (%)	17 (25.0)	21 (25.6)	1.000

**FIGURE 3:**
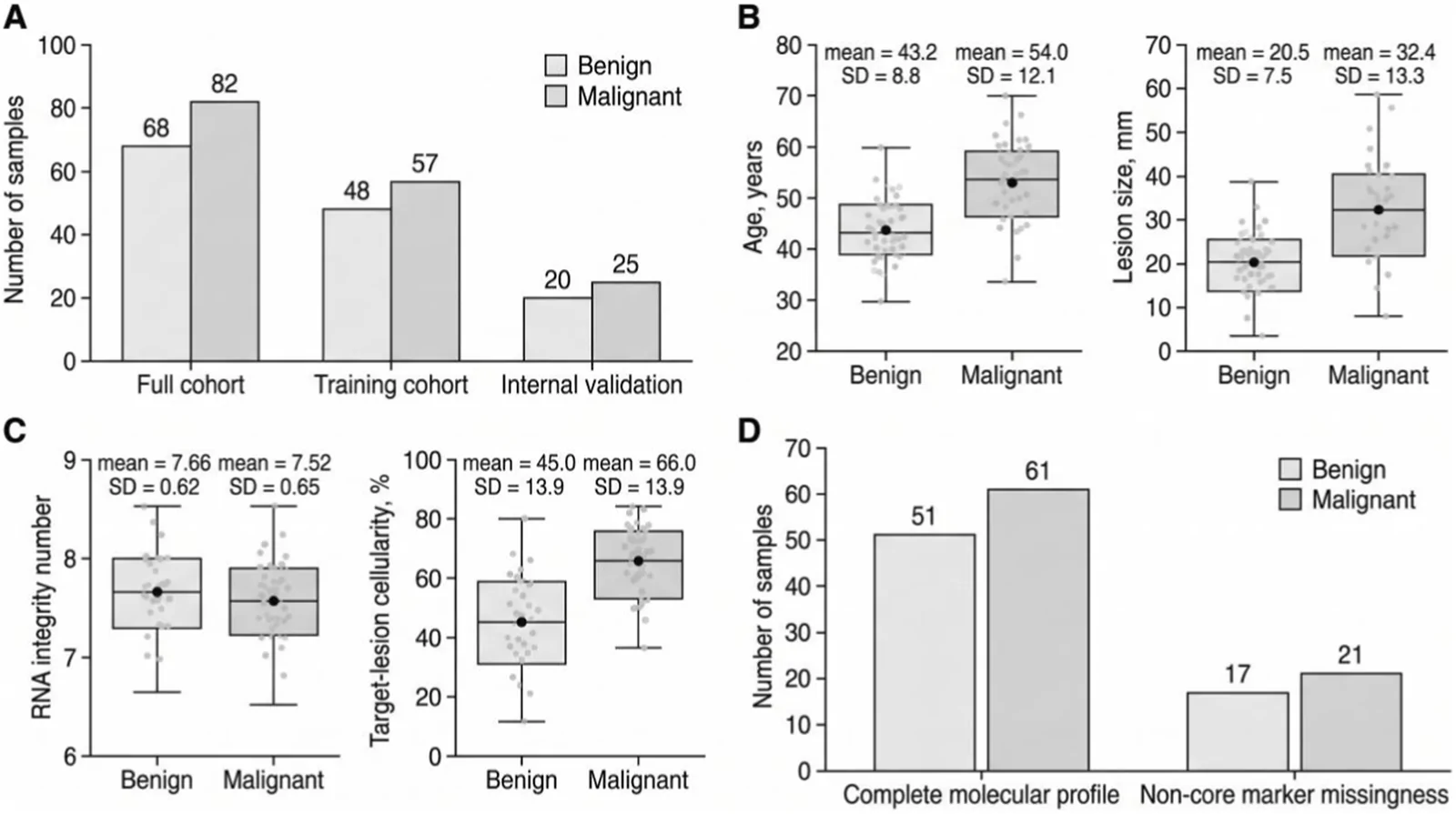
Tissue cohort characteristics and RT-qPCR quality statistics. **(A)** Diagnostic-group distribution in the full cohort, training cohort, and internal-validation cohort. **(B)** Age and lesion-size distributions by diagnostic group. **(C)** RNA integrity number and target-lesion cellularity distributions. **(D)** Missingness pattern for core and non-core molecular markers. Abbreviations: RT-qPCR, reverse-transcription quantitative polymerase chain reaction; RNA, ribonucleic acid; SD, standard deviation.

Values are mean ± SD or n (%). BI-RADS categories were compared using one overall chi-square test (χ^2^ = 67.54, df = 2, *P* < 0.001). Estimated malignant-minus-benign differences (95% CI) were: age 10.8 years (7.4–14.2), postmenopausal prevalence 26.0 percentage points (10.9–41.0), lesion size 11.9 mm (8.5–15.3), RNA integrity number −0.14 (−0.35 to 0.07), target-lesion cellularity 21.0 percentage points (16.5–25.5), complete molecular profile −0.6 percentage points (−14.6 to 13.4), and non-core marker missingness 0.6 percentage points (−13.4–14.6).

### 3.3 RT-qPCR evaluation of candidate inverse miRNA–mRNA axes

The three prespecified core axes showed the expected malignant-associated expression direction. miR-205-5p was lower and HMGB3 higher in malignant tumors; miR-96-5p and miR-21-5p were higher, whereas FOXO1 and PIK3R1 were lower. In pooled descriptive correlation testing, miR-205-5p/HMGB3 and miR-96-5p/FOXO1 exceeded the prespecified inverse-correlation magnitude threshold. miR-21-5p/PIK3R1 showed the expected inverse expression direction but did not reach r < −0.20. Because pooled correlations can be influenced by diagnostic-group separation, these findings are reported as candidate inverse-expression evidence rather than direct regulatory validation.

Among the network-supported axes, miR-182-5p/ARRDC3, miR-200c-3p/FBLN5, miR-342-3p/TACC1, and miR-125b-5p/PARP1 met the prespecified inverse-correlation magnitude criterion and remained significant after Benjamini–Hochberg correction. miR-195-5p/RACGAP1, miR-139-5p/TPD52, and miR-145-5p/TPM3 showed the expected expression direction but did not meet the complete inverse-candidate rule. Candidate-axis summary statistics are presented in Table 2, revised Figure 4, and Supplementary Table S7. The relative ordering of the ten measured axes by absolute pooled correlation and adjusted significance is provided in Supplementary Table S2.

**TABLE 2:** Candidate inverse miRNA–mRNA axes in the RT-qPCR cohort; correlations are pooled descriptive statistics.

miRNA–mRNA axis	Pattern in malignant tumors	Benign miRNA mean	Malignant miRNA mean	Benign mRNA mean	Malignant mRNA mean	Pearson r	Raw P/BH-adjusted P	Result classification
miR-205-5p/HMGB3	Down-miRNA/up-mRNA	6.873	5.897	4.865	6.083	−0.345	<0.001/<0.003	Core inverse-expression candidate
miR-96-5p/FOXO1	Up-miRNA/down-mRNA	5.342	6.170	6.775	5.864	−0.354	<0.001/<0.003	Core inverse-expression candidate
miR-21-5p/PIK3R1	Up-miRNA/down-mRNA	6.171	6.914	6.466	5.742	−0.185	0.023/0.029	Directional core marker
miR-182-5p/ARRDC3	Up-miRNA/down-mRNA	5.475	6.221	6.184	5.680	−0.497	<0.001/<0.003	Network-supported inverse candidate
miR-200c-3p/FBLN5	Up-miRNA/down-mRNA	6.079	6.740	6.391	5.813	−0.206	0.014/0.023	Network-supported inverse candidate
miR-342-3p/TACC1	Up-miRNA/down-mRNA	5.844	6.566	5.497	4.968	−0.257	0.002/0.004	Network-supported inverse candidate
miR-195-5p/RACGAP1	Down-miRNA/up-mRNA	6.055	5.426	5.587	5.792	−0.188	0.026/0.029	Exploratory measured axis
miR-139-5p/TPD52	Down-miRNA/up-mRNA	5.606	5.057	5.883	6.590	−0.199	0.017/0.024	Exploratory measured axis
miR-125b-5p/PARP1	Down-miRNA/up-mRNA	5.997	5.633	5.872	6.263	−0.283	<0.001/<0.003	Network-supported inverse candidate
miR-145-5p/TPM3	Down-miRNA/up-mRNA	5.987	5.434	6.287	6.658	−0.155	0.065/0.065	Exploratory measured axis

**FIGURE 4:**
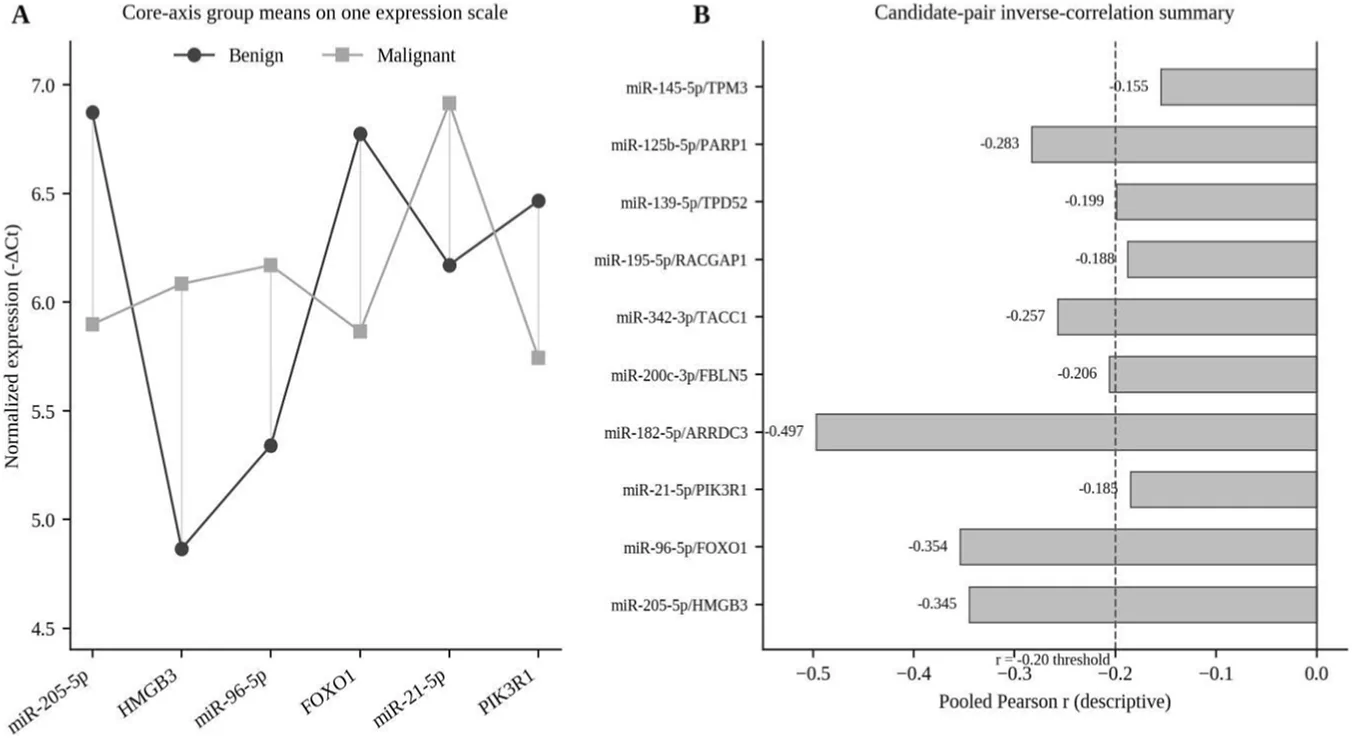
RT-qPCR expression and descriptive inverse-correlation statistics. **(A)** Group means for the six core-axis markers on the normalized −ΔCt scale. **(B)** Pooled Pearson r values for the ten candidate pairs; the dashed line marks r = −0.20. These correlations are descriptive and do not establish direct regulation. Abbreviations: RT-qPCR, reverse-transcription quantitative polymerase chain reaction; Ct, cycle threshold; ΔCt, difference between target and reference Ct values.

In the supplementary correlation analysis, miR-182-5p/ARRDC3 retained inverse associations in both benign (r = −0.492, BH q < 0.001) and malignant (r = −0.441, BH q < 0.001) groups and after adjustment for diagnosis, age, and cellularity (partial r = −0.460, BH q < 0.001). miR-96-5p/FOXO1 remained significant in malignant samples (r = −0.345, BH q = 0.008) and in the adjusted pooled analysis (partial r = −0.231, BH q = 0.017). miR-125b-5p/PARP1 also retained adjusted inverse evidence (partial r = −0.244, BH q = 0.016). Several other pooled inverse correlations weakened after within-group and covariate adjustment. These supplementary results support cautious candidate-pair terminology and are analytically separate from the primary pooled correlation results.

### 3.4 Diagnostic model development and internal validation

LASSO logistic regression selected five molecular predictors: miR-205-5p, HMGB3, miR-96-5p, FOXO1, and miR-21-5p. All five retained markers had complete expression measurements and were therefore used without imputation in the refitted model and internal-validation score. Lower miR-205-5p and FOXO1 values were associated with higher malignant probability, whereas higher HMGB3, miR-96-5p, and miR-21-5p values were associated with higher malignant probability. Using standardized marker values, the refitted linear predictor was: 0.18–1.12×miR-205-5p + 1.03×HMGB3 + 0.88×miR-96-5p − 0.91×FOXO1 + 0.74×miR-21-5p. The locked malignant-classification threshold was 0.52.

The locked five-marker score used the coefficients shown above and the prespecified 0.52 threshold. Supplementary Table S8 provides the corresponding standardization parameters used in the supplementary analysis. Repeated complete-case full-panel screening is summarized separately in Supplementary Table S5 because the broader full-panel selection pattern included several additional variables and therefore should not be interpreted as a replacement for the original five-marker selection result.

The training cohort AUC was 0.938 (95% CI 0.891–0.979). In the internal-validation cohort, AUC was 0.894 (0.788–0.975), accuracy 0.844 (0.712–0.923), sensitivity 0.840 (0.653–0.936), specificity 0.850 (0.640–0.948), PPV 0.875 (0.690–0.957), and NPV 0.810 (0.600–0.923). The pooled cohort performance is reported descriptively and was not used as an independent validation estimate. Model discrimination and classification statistics are shown in Table 3 and Figure 5. Detailed classification metrics with 95% confidence intervals are provided in Supplementary Table S9.

**TABLE 3:** Diagnostic-model performance.

Cohort	n	AUC (95% CI)	Accuracy (95% CI)	Sensitivity (95% CI)	Specificity (95% CI)	PPV (95% CI)	NPV (95% CI)	F1	TN/FP/FN/TP
Training	105	0.938 (0.891–0.979)	0.829 (0.745–0.889)	0.772 (0.648–0.862)	0.896 (0.778–0.955)	0.898 (0.782–0.956)	0.768 (0.642–0.859)	0.830	43/5/13/44
Internal validation	45	0.894 (0.788–0.975)	0.844 (0.712–0.923)	0.840 (0.653–0.936)	0.850 (0.640–0.948)	0.875 (0.690–0.957)	0.810 (0.600–0.923)	0.857	17/3/4/21
Pooled cohort, descriptive only	150	0.922 (0.875–0.957)	0.833 (0.766–0.884)	0.793 (0.693–0.866)	0.882 (0.785–0.939)	0.890 (0.798–0.943)	0.779 (0.675–0.857)	0.839	60/8/17/65

**FIGURE 5:**
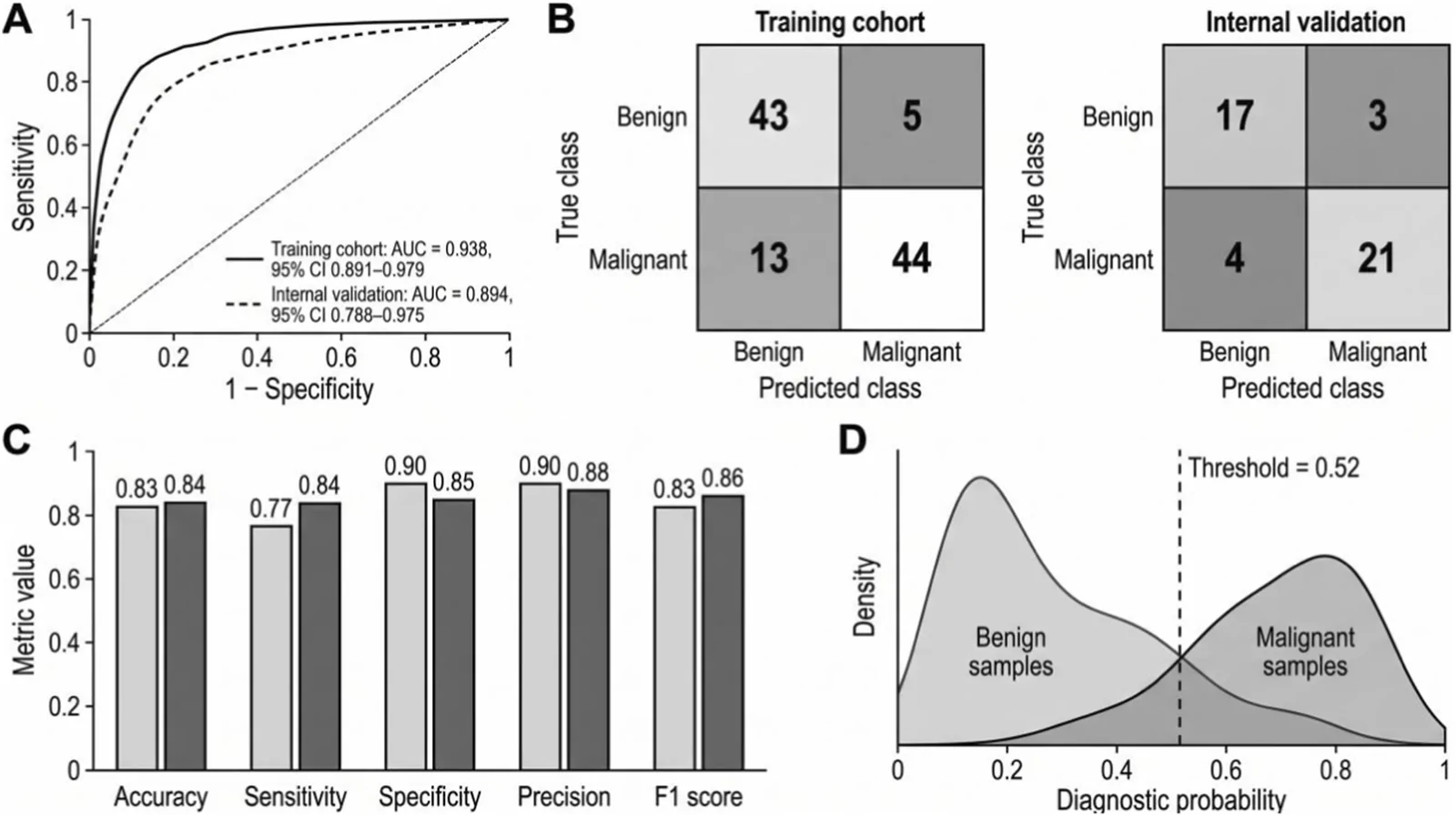
Diagnostic-model performance. **(A)** Receiver-operating-characteristic curves for the training and internal-validation cohorts. **(B)** Confusion matrices at the locked 0.52 threshold. **(C)** Classification metrics by cohort. **(D)** Diagnostic-probability distributions in benign and malignant samples. Abbreviations: AUC, area under the receiver operating characteristic curve; CI, confidence interval; F1, F1 score.

### 3.5 Calibration, decision-curve analysis, and sensitivity analyses

In the internal-validation cohort, the calibration intercept was −0.07, the calibration slope was 0.89, and the Brier score was 0.151. Decision-curve analysis showed higher net benefit than treat-all and treat-none strategies across thresholds of 0.20–0.75. Sensitivity-analysis AUCs were 0.872 after age/BI-RADS adjustment, 0.886 after excluding ductal carcinoma *in situ*, and 0.902 in the RIN≥6.0/cellularity≥40% subset. Across 100 repeated splits, median AUC was 0.887 (interquartile range [IQR] 0.854–0.918). Details are shown in Figure 6 and Supplementary Table S10.

**FIGURE 6:**
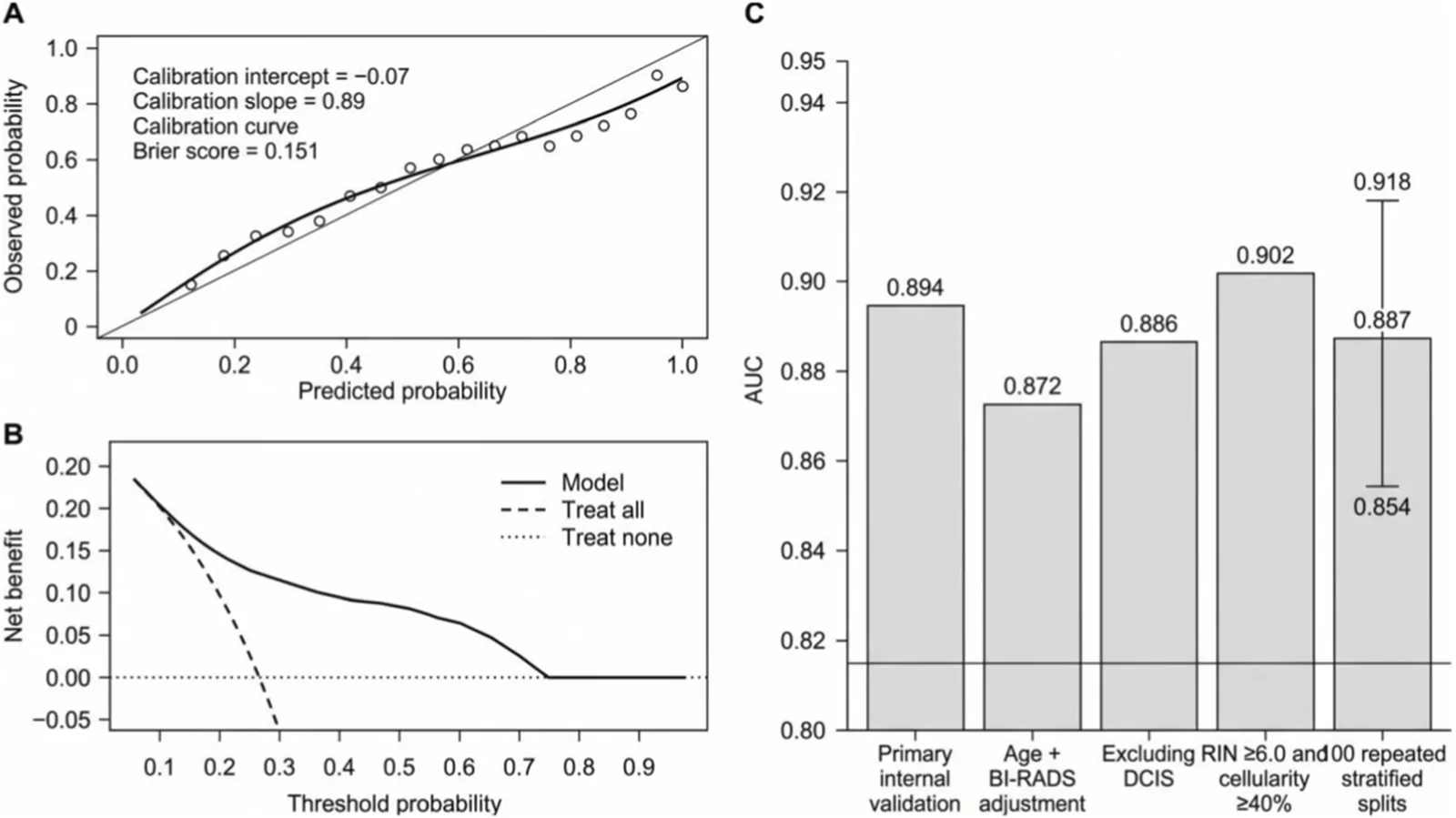
Calibration, decision-curve, and sensitivity statistics. **(A)** Calibration plot for the internal-validation cohort. **(B)** Decision-curve analysis across threshold probabilities from 0.10 to 0.90. **(C)** Sensitivity-analysis AUC values under clinical adjustment, exclusion of ductal carcinoma *in situ*, stricter RNA-quality thresholds, and repeated train-validation splitting. Abbreviations: AUC, area under the receiver operating characteristic curve; BI-RADS, Breast Imaging Reporting and Data System; DCIS, ductal carcinoma *in situ*; RIN, RNA integrity number; RNA, ribonucleic acid.

### 3.6 Supplementary robustness analyses

To avoid mixing distinct analysis layers, the prespecified repeated-split result remains the primary estimate reported in Section 3.5 (median AUC 0.887, IQR 0.854–0.918). A separate supplementary stability analysis yielded a median validation AUC of 0.918 (IQR 0.890–0.936; 2.5th–97.5th percentile 0.848–0.974); this value is therefore not substituted for the primary repeated-split estimate.

In the supplementary model-comparison analysis, AUCs in the fixed 45-record validation partition were 0.835 for BI-RADS alone, 0.914 for the clinical model, 0.900 for the molecular score, and 0.966 for the combined clinical–molecular model, with corresponding Brier scores of 0.147, 0.119, 0.132, and 0.086. These estimates are interpreted separately from the primary internal-validation AUC of 0.894 reported in Section 3.4.

The supplementary BI-RADS four analysis included 70 records (28 benign and 42 malignant). The molecular score yielded an AUC of 0.921 (bootstrap 95% CI 0.849–0.974) in the overall BI-RADS four subset and 0.905 (95% CI 0.762–1.000) in the validation-partition subset (n = 23; nine benign and 14 malignant), with eight true negatives, one false positive, three false negatives, and 11 true positives at the 0.52 threshold. Because the validation subgroup is small, this estimate is exploratory.

For calibration, the primary internal-validation estimates remain the prespecified values reported in Section 3.5 (intercept −0.07, slope 0.89, Brier score 0.151). The supplementary analysis yielded intercept 0.800 (95% CI −0.319 to 1.875), slope 0.750 (95% CI 0.481–1.527), and Brier score 0.132 (95% CI 0.066–0.207); these uncertainty estimates are provided only as a supplementary robustness check and do not supersede the primary calibration results. Full numerical outputs are provided in Supplementary Tables S5, 6, 8, 11-14.

## 4 Discussion

This study identified candidate inverse miRNA–mRNA expression patterns in breast lesions and developed a five-marker tissue-based diagnostic model. miR-205-5p/HMGB3 and miR-96-5p/FOXO1 provided the strongest inverse-expression evidence, whereas miR-21-5p contributed mainly as a directional marker. In internal validation, the model achieved an AUC of 0.894, accuracy of 0.844, sensitivity of 0.840, specificity of 0.850, and acceptable calibration (Shannon et al., 2003).

The miR-205-5p/HMGB3 axis was one of the clearest candidates. miR-205-5p was lower and HMGB3 higher in malignant tissue, consistent with evidence that miR-205 has tumor-suppressive functions and that HMGB3 is associated with malignant behavior in breast cancer (Wu et al., 2009; Elgamal et al., 2013). The reciprocal expression pattern, target-resource support, and pooled inverse association make this axis a high-priority candidate for functional validation, although the present data do not prove direct repression (Steyerberg, 2019; Van Calster et al., 2019).

The miR-96-5p/FOXO1 axis showed a similarly coherent pattern. miR-96-5p was higher and FOXO1 lower in malignant tissue; prior cell-based work links miR-96 to FOXO1 repression, while FOXO1 has tumor-suppressive activity in breast cancer (Guttilla and White, 2009). The agreement between expression direction, target support, and tissue-level inverse association supports prioritization of this axis for reporter and protein-level validation (Siddiqui et al., 2026).

The miR-21-5p/PIK3R1 finding illustrates the importance of separating diagnostic contribution from pair-level interpretation. miR-21-5p was higher and PIK3R1 lower in malignant tumors, consistent with prior breast-cancer evidence involving miR-21 and PI3K/AKT-related signaling (Yan et al., 2016), but the pooled correlation did not cross the prespecified r < −0.20 magnitude threshold. miR-21-5p was therefore retained as a model-selected directional marker, whereas the miR-21-5p/PIK3R1 pair was not classified as a supported inverse-expression candidate.

Other network-supported axes, including miR-182-5p/ARRDC3, miR-200c-3p/FBLN5, miR-342-3p/TACC1, and miR-125b-5p/PARP1, also showed inverse-expression evidence after multiplicity correction. These findings are secondary candidates because the study did not include reporter assays, protein validation, knockdown, or overexpression experiments (Hayes et al., 2014; Zou et al., 2022).

The diagnostic model showed stable primary performance after training-cohort marker selection and locked-threshold validation. The final model retained five molecular predictors and was applied to the internal-validation cohort without marker reselection, coefficient refitting, threshold re-optimization, or normalization recalibration. Supplementary analyses further examined a BI-RADS four subgroup and incremental-value comparison. These estimates are presented as exploratory robustness information rather than as replacements for the primary clinical performance metrics.

Several limitations remain. Public datasets were used mainly for tumor-associated discovery rather than as a complete benign–malignant discovery cohort, and pooled miRNA–mRNA correlations may partly reflect between-group separation. The study was single-center, the internal-validation cohort was small, and target-lesion cellularity differed between diagnostic groups. The supplementary robustness estimates in Supplementary Tables S5, 6, 8, 11-14 should be confirmed against the original patient-level source dataset before being interpreted as observed clinical results. In addition, the Supplementary Material reports the measured-axis analyses and public-source context for the ten validated axes; the full 36-pair public-discovery ranking and complete cytoHubba/MCODE and GO/KEGG output tables are not included. External validation and functional confirmation remain necessary before clinical translation (McShane et al., 2013). Public-source TCGA correlation context for the ten measured axes is provided in Supplementary Table S15.

## 5 Conclusion

This study identified candidate inverse miRNA–mRNA expression features associated with malignant breast lesions and developed a five-marker tissue-based model with promising internal discrimination. The strongest inverse-expression evidence involved miR-205-5p/HMGB3 and miR-96-5p/FOXO1. The model should be considered an adjunct to pathological assessment; independent validation and functional confirmation are required before clinical application.

## Data Availability

The original contributions presented in the study are included in the article/Supplementary Material, further inquiries can be directed to the corresponding author.
