# Supplementary material for: Identification of candidate inverse miRNA–mRNA pairs and an internally validated tissue-based diagnostic model for benign and malignant breast lesions
Source: Front Mol Biosci. 2026 Oct 23;13:1952773. doi: 10.3389/fmolb.2026.1952773 (PMC13739551; doi:10.3389/fmolb.2026.1952773)
Supplement: Supplementary file 1 [file Table1.docx]

Supplementary Material

# Supplementary Tables

**Supplementary Table 1.** Public discovery datasets, target-interaction resources, and analysis modules used for candidate-pair screening.

| **Module** | **Locked item** | **Platform / version** | **Sample scope** | **Role** | **Output / source** |
| --- | --- | --- | --- | --- | --- |
| miRNA discovery 1 | GSE45666 | Agilent human miRNA microarray; GPL14767 | 101 breast tumors; 15 adjacent normal tissues | Cancer-associated miRNA direction screen | Differential miRNA list |
| miRNA discovery 2 | GSE26659 | Agilent human miRNA platform V2; GPL8227 | 77 ductal breast carcinoma biopsies; 17 mammoplasty normal tissues | Cross-dataset miRNA direction check | Replicated miRNA direction |
| miRNA tumor-network support | GSE58210 | Agilent Human miRNA Microarray Kit release 14.0 V2; design ID 029297 | 283 breast tumors | Tumor-level miRNA expression support | Tumor miRNA expression matrix |
| Benign–malignant direction check | GSE144534 | Affymetrix GeneChip miRNA 4.0 Array | FFPE breast tissues, including benign non-cancer and malignant samples | Secondary tissue-level miRNA direction check | Candidate miRNA benign–malignant direction support |
| mRNA discovery 1 | GSE109169 | Affymetrix Human Exon 1.0 ST Array; GPL5175 | 25 paired normal/tumor breast tissue sets | Primary mRNA direction screen | Differential mRNA list |
| mRNA discovery 2 | GSE139038 | Two-dye cDNA microarray; GPL27630 | 41 tumors; 18 paired normal tissues; 6 apparently normal non-malignant tissues | mRNA direction replication | Replicated mRNA direction |
| Paired correlation | TCGA-BRCA | GDC miRNA-seq and mRNA expression data | Tumor-level paired expression data; pair-wise complete-case intersection of available miRNA and mRNA measurements | miRNA–mRNA correlation analysis only; not a benign comparator | Pearson correlation matrix for paired tumor samples; pair-specific n not reported in retained source files |
| miRNA nomenclature | miRBase | Release 22.1; mature.fa | Homo sapiens mature miRNA names | Identifier harmonization | Official mature miRNA name table |
| Validated target database | miRTarBase | 2025 release | Experimentally supported human miRNA–mRNA interactions | First-priority target filter | Validated target-pair list |
| Predicted target database | TargetScanHuman | 8.0 | Human 3′ UTR seed-match prediction | Seed-region support | Predicted target list |
| Predicted target database | miRDB | 6.0 | Human predicted targets | Functional target-prediction support | Predicted target list |
| CLIP-supported database | ENCORI/starBase | 3.0 | Ago/CLIP-supported RNA interaction evidence | CLIP-backed target support | CLIP-supported candidate pairs |
| Data retrieval | GEOquery | 2.74.0; R 4.4.2 | GEO matrix and platform files | Data import | ExpressionSet objects |
| Preprocessing | preprocessCore | 1.68.0; R 4.4.2 | Microarray expression matrices | Quantile normalization | Normalized matrices |
| Differential expression | limma | 3.62.2; R 4.4.2 | Dataset-specific expression matrices | Empirical Bayes linear modeling | log2 fold change, *P* value, and adjusted *P* value |
| Batch visualization | sva / ComBat | sva 3.54.0; R 4.4.2 | Cross-platform expression matrices | Exploratory visualization only | Batch-adjusted exploratory matrix |
| Network construction | Cytoscape | 3.10.3 | Retained inverse miRNA–mRNA pairs | Directed network construction | Node and edge tables |
| Hub ranking | cytoHubba | 0.1 | Cytoscape network | Degree and betweenness ranking | Hub miRNAs and mRNAs |
| Module detection | MCODE | 2.0.3 | Cytoscape network | Local module detection | Network modules |
| Pathway annotation | clusterProfiler / org.Hs.eg.db | clusterProfiler 4.14.4; org.Hs.eg.db 3.20.0 | Candidate mRNA list | GO and KEGG enrichment | Enriched pathway table |
| Diagnostic modeling | glmnet / caret / pROC / rms / rmda / mice | glmnet 4.1-8; caret 6.0-94; pROC 1.18.5; rms 6.8-1; rmda 1.6; mice 3.16.0 | RT-qPCR validation cohort | LASSO, logistic regression, ROC analysis, calibration, decision-curve analysis, and imputation | Diagnostic probability and validation metrics |

**Supplementary Table 2.** Relative ranking and reporting characteristics of the ten RT-qPCR candidate inverse miRNA–mRNA axes.

| **Rank** | **Axis** | **Malignant pattern** | **\|r\|** | **Raw *P* / BH *P*** | **Panel classification** |
| --- | --- | --- | --- | --- | --- |
| 1 | miR-182-5p/ARRDC3 | Up-miRNA/down-mRNA | 0.497 | <0.001 / <0.003 | Network-supported inverse candidate |
| 2 | miR-96-5p/FOXO1 | Up-miRNA/down-mRNA | 0.354 | <0.001 / <0.003 | Core inverse-expression candidate |
| 3 | miR-205-5p/HMGB3 | Down-miRNA/up-mRNA | 0.345 | <0.001 / <0.003 | Core inverse-expression candidate |
| 4 | miR-125b-5p/PARP1 | Down-miRNA/up-mRNA | 0.283 | <0.001 / <0.003 | Network-supported inverse candidate |
| 5 | miR-342-3p/TACC1 | Up-miRNA/down-mRNA | 0.257 | 0.002 / 0.004 | Network-supported inverse candidate |
| 6 | miR-200c-3p/FBLN5 | Up-miRNA/down-mRNA | 0.206 | 0.014 / 0.023 | Network-supported inverse candidate |
| 7 | miR-139-5p/TPD52 | Down-miRNA/up-mRNA | 0.199 | 0.017 / 0.024 | Exploratory measured axis |
| 8 | miR-195-5p/RACGAP1 | Down-miRNA/up-mRNA | 0.188 | 0.026 / 0.029 | Exploratory measured axis |
| 9 | miR-21-5p/PIK3R1 | Up-miRNA/down-mRNA | 0.185 | 0.023 / 0.029 | Directional core marker |
| 10 | miR-145-5p/TPM3 | Down-miRNA/up-mRNA | 0.155 | 0.065 / 0.065 | Exploratory measured axis |

Note: Ranking is descriptive and based on the absolute pooled RT-qPCR correlation magnitude among the ten measured axes. Raw and Benjamini–Hochberg-adjusted *P* values are reproduced from Supplementary Table 7. Pair-specific public-discovery counts and the complete 36-pair ranking were not available in the retained source files.

**Supplementary Table 3.** Clinical variables, molecular variables, quality-control thresholds, and analytic roles.

| **Variable name** | **Type** | **Coding / unit** | **Source or QC rule** | **Analytic role** |
| --- | --- | --- | --- | --- |
| sample_id | Identifier | BT001–BT150 | Study database; not missing | Unique sample identifier |
| collection_date | Date | 6 January 2025 to 31 October 2025 | Pathology or laboratory record | Sampling chronology |
| diagnosis_group | Categorical | Benign; Malignant | Final pathology report; not missing | Reference diagnosis |
| malignant_status | Binary | 0 = benign; 1 = malignant | Derived from diagnosis_group | Model outcome |
| cohort_split | Categorical | Training; Internal validation | Diagnosis-stratified randomization; seed 20251115 | Model development and validation |
| age_years | Continuous | Years | Clinical record | Clinical covariate |
| menopausal_status | Categorical | Premenopausal; Postmenopausal; Unknown | Clinical record | Descriptive covariate |
| BI_RADS | Ordinal | 2, 3, 4A, 4B, 4C, 5 | Imaging report closest to tissue sampling | Clinical-adjustment covariate |
| lesion_size_mm | Continuous | Largest diameter, mm | Imaging or pathology report | Descriptive and sensitivity covariate |
| histology | Categorical | Fibroadenoma; fibrocystic change; adenosis; intraductal papilloma; benign phyllodes tumor; ductal carcinoma in situ; invasive ductal carcinoma; invasive lobular carcinoma; other carcinoma | Final pathology report | Histological subgroup |
| ER_status | Categorical | Positive; Negative; Not applicable | Positive if ≥1% nuclear staining | Malignant-tumor descriptor |
| PR_status | Categorical | Positive; Negative; Not applicable | Positive if ≥1% nuclear staining | Malignant-tumor descriptor |
| HER2_status | Categorical | Positive; Negative; Not applicable; missing if unresolved IHC 2+ without ISH | IHC or ISH report | Malignant-tumor descriptor |
| Ki67_percent | Continuous | % positive tumor nuclei | Immunohistochemistry report | Malignant-tumor descriptor |
| target_lesion_cellularity_percent | Continuous | % lesion cells | H&E review; exclude if <30% | Tissue adequacy QC |
| RIN | Continuous | RNA integrity number | Agilent 2100 Bioanalyzer; exclude if <5.5 | RNA quality QC |
| A260_A280 | Continuous | Ratio | NanoDrop 2000; repeat extraction if outside 1.80–2.10 | RNA purity QC |
| A260_A230 | Continuous | Ratio | NanoDrop 2000; repeat extraction if <1.70 | RNA purity QC |
| RNA_input_ng | Continuous | ng | Qubit RNA HS Assay; exclude if <100 ng after repeat extraction | RNA input QC |
| miR_205_5p_expr | Continuous | −ΔCt | RT-qPCR; core-axis marker; not imputed | Molecular predictor |
| HMGB3_expr | Continuous | −ΔCt | RT-qPCR; core-axis marker; not imputed | Molecular predictor |
| miR_96_5p_expr | Continuous | −ΔCt | RT-qPCR; core-axis marker; not imputed | Molecular predictor |
| FOXO1_expr | Continuous | −ΔCt | RT-qPCR; core-axis marker; not imputed | Molecular predictor |
| miR_21_5p_expr | Continuous | −ΔCt | RT-qPCR; core-axis marker; not imputed | Molecular predictor |
| PIK3R1_expr | Continuous | −ΔCt | RT-qPCR; core-axis paired target; not imputed for pair-level validation | Molecular predictor for axis validation |
| miR_182_5p_expr | Continuous | −ΔCt | RT-qPCR; candidate-axis marker | Candidate predictor |
| ARRDC3_expr | Continuous | −ΔCt | RT-qPCR; candidate-axis paired target | Candidate predictor |
| miR_200c_3p_expr | Continuous | −ΔCt | RT-qPCR; candidate-axis marker | Candidate predictor |
| FBLN5_expr | Continuous | −ΔCt | RT-qPCR; candidate-axis paired target | Candidate predictor |
| miR_342_3p_expr | Continuous | −ΔCt | RT-qPCR; candidate-axis marker | Candidate predictor |
| TACC1_expr | Continuous | −ΔCt | RT-qPCR; candidate-axis paired target | Candidate predictor |
| miR_195_5p_expr | Continuous | −ΔCt | RT-qPCR; exploratory-axis marker | Exploratory predictor |
| RACGAP1_expr | Continuous | −ΔCt | RT-qPCR; exploratory-axis paired target | Exploratory predictor |
| miR_139_5p_expr | Continuous | −ΔCt | RT-qPCR; exploratory-axis marker | Exploratory predictor |
| TPD52_expr | Continuous | −ΔCt | RT-qPCR; exploratory-axis paired target | Exploratory predictor |
| miR_125b_5p_expr | Continuous | −ΔCt | RT-qPCR; candidate-axis marker | Candidate predictor |
| PARP1_expr | Continuous | −ΔCt | RT-qPCR; candidate-axis paired target | Candidate predictor |
| miR_145_5p_expr | Continuous | −ΔCt | RT-qPCR; exploratory-axis marker | Exploratory predictor |
| TPM3_expr | Continuous | −ΔCt | RT-qPCR; exploratory-axis paired target | Exploratory predictor |
| miRNA_reference_mean | Continuous | Geometric mean Ct of hsa-miR-16-5p and hsa-miR-191-5p | Required for miRNA normalization | miRNA normalization denominator |
| mRNA_reference_mean | Continuous | Geometric mean Ct of GAPDH and ACTB | Required for mRNA normalization | mRNA normalization denominator |
| technical_replicate_SD | Continuous | Ct SD across technical triplicates | Repeat assay if >0.35 | Assay repeat trigger |
| failed_Ct_flag | Binary | 1 = Ct >35, undetermined Ct, or NTC amplification before Ct 38 | Failed target recorded as missing after repeat failure | Assay QC exclusion |
| interplate_drift_Ct | Continuous | Ct drift | Repeat plate if drift >0.50 Ct | Plate-level QC |
| diagnostic_probability | Continuous | 0–1 | Logistic-regression output | Diagnostic-model output |
| predicted_class | Binary | 0 = benign; 1 = malignant | diagnostic_probability ≥0.52 | Locked classification output |

**Supplementary Table 4.** RT-qPCR assay, reagent, instrument, and reproducibility information.

| **Category** | **Target / item** | **Product / assay** | **Manufacturer** | **Catalog number / assay ID** | **QC / use rule** |
| --- | --- | --- | --- | --- | --- |
| RNA extraction | Small-RNA-containing total RNA | mirVana miRNA Isolation Kit, with phenol | Invitrogen / Thermo Fisher Scientific | AM1560 | Used for all validation tissues; small-RNA-containing total RNA extraction |
| RNA quantification | RNA concentration | Qubit RNA HS Assay Kit | Invitrogen / Thermo Fisher Scientific | Q32852 | RNA input accepted when total input was ≥100 ng |
| RNA purity | A260/A280 and A260/A230 | NanoDrop 2000 Spectrophotometer | Thermo Scientific | ND-2000 | A260/A280 accepted at 1.80–2.10; A260/A230 accepted at ≥1.70 |
| RNA integrity | RNA integrity number | Agilent 2100 Bioanalyzer with RNA 6000 Nano Kit | Agilent | 5067-1511 | RIN ≥5.5 required for primary inclusion; RIN ≥6.0 used for high-quality RNA sensitivity analysis |
| miRNA reverse transcription | Mature miRNA cDNA synthesis | TaqMan Advanced miRNA cDNA Synthesis Kit | Applied Biosystems / Thermo Fisher Scientific | A28007 | Same miRNA cDNA synthesis workflow used for all miRNA assays |
| miRNA qPCR assay format | Mature miRNA assays | TaqMan Advanced miRNA Assay | Applied Biosystems / Thermo Fisher Scientific | A25576 + target-specific assay ID | Probe-based miRNA quantification; target-specific assay IDs listed below |
| miRNA qPCR master mix | miRNA qPCR amplification | TaqMan Fast Advanced Master Mix for qPCR | Applied Biosystems / Thermo Fisher Scientific | 4444557 | Same master mix used across all miRNA plates |
| mRNA reverse transcription | mRNA cDNA synthesis | High-Capacity cDNA Reverse Transcription Kit | Applied Biosystems / Thermo Fisher Scientific | 4368814 | Same reverse-transcription workflow used for mRNA targets and reference genes |
| mRNA qPCR master mix | SYBR-based mRNA qPCR | PowerUp SYBR Green Master Mix | Applied Biosystems / Thermo Fisher Scientific | A25742 | Single melt-curve peak required for every accepted mRNA target |
| qPCR instrument | Real-time PCR | QuantStudio 5 Real-Time PCR System, 96-well, 0.2 mL, desktop | Applied Biosystems / Thermo Fisher Scientific | A28574 | Each target measured in technical triplicate |
| qPCR reporting | Reporting standard | MIQE guidelines | Clinical Chemistry / MIQE | PMID: 19246619 | Ct values, controls, normalization, assay QC, and repeat rules reported |
| miRNA assay | hsa-miR-205-5p | TaqMan Advanced miRNA Assay | Applied Biosystems / Thermo Fisher Scientific | 477967_mir | Candidate miRNA for HMGB3 axis; Ct ≤35 and triplicate SD ≤0.35 required |
| miRNA assay | hsa-miR-96-5p | TaqMan Advanced miRNA Assay | Applied Biosystems / Thermo Fisher Scientific | 478215_mir | Candidate miRNA for FOXO1 axis; Ct ≤35 and triplicate SD ≤0.35 required |
| miRNA assay | hsa-miR-21-5p | TaqMan Advanced miRNA Assay | Applied Biosystems / Thermo Fisher Scientific | 477975_mir | Candidate miRNA for PIK3R1 axis; Ct ≤35 and triplicate SD ≤0.35 required |
| miRNA assay | hsa-miR-182-5p | TaqMan Advanced miRNA Assay | Applied Biosystems / Thermo Fisher Scientific | 477935_mir | Candidate miRNA for ARRDC3 axis; Ct ≤35 and triplicate SD ≤0.35 required |
| miRNA assay | hsa-miR-200c-3p | TaqMan Advanced miRNA Assay | Applied Biosystems / Thermo Fisher Scientific | 478351_mir | Candidate miRNA for FBLN5 axis; Ct ≤35 and triplicate SD ≤0.35 required |
| miRNA assay | hsa-miR-342-3p | TaqMan Advanced miRNA Assay | Applied Biosystems / Thermo Fisher Scientific | 478043_mir | Candidate miRNA for TACC1 axis; Ct ≤35 and triplicate SD ≤0.35 required |
| miRNA assay | hsa-miR-195-5p | TaqMan Advanced miRNA Assay | Applied Biosystems / Thermo Fisher Scientific | 477957_mir | Candidate miRNA for RACGAP1 axis; Ct ≤35 and triplicate SD ≤0.35 required |
| miRNA assay | hsa-miR-139-5p | TaqMan Advanced miRNA Assay | Applied Biosystems / Thermo Fisher Scientific | 478312_mir | Candidate miRNA for TPD52 axis; Ct ≤35 and triplicate SD ≤0.35 required |
| miRNA assay | hsa-miR-125b-5p | TaqMan Advanced miRNA Assay | Applied Biosystems / Thermo Fisher Scientific | 477885_mir | Candidate miRNA for PARP1 axis; Ct ≤35 and triplicate SD ≤0.35 required |
| miRNA assay | hsa-miR-145-5p | TaqMan Advanced miRNA Assay | Applied Biosystems / Thermo Fisher Scientific | 477916_mir | Candidate miRNA for TPM3 axis; Ct ≤35 and triplicate SD ≤0.35 required |
| miRNA reference | hsa-miR-16-5p | TaqMan Advanced miRNA Assay | Applied Biosystems / Thermo Fisher Scientific | 477860_mir | Used in geometric mean with hsa-miR-191-5p for miRNA normalization |
| miRNA reference | hsa-miR-191-5p | TaqMan Advanced miRNA Assay | Applied Biosystems / Thermo Fisher Scientific | 477952_mir | Used in geometric mean with hsa-miR-16-5p for miRNA normalization |
| mRNA assay | HMGB3 | qSTAR qPCR Primer Pair | OriGene Technologies | HP208497; NM_005342 | Paired mRNA target for miR-205-5p; single melt-curve peak required |
| mRNA assay | FOXO1 | qSTAR qPCR Primer Pair | OriGene Technologies | HP205770; NM_002015 | Paired mRNA target for miR-96-5p; single melt-curve peak required |
| mRNA assay | PIK3R1 | qSTAR qPCR Primer Pair | OriGene Technologies | HP225728; NM_181523 | Paired mRNA target for miR-21-5p; single melt-curve peak required |
| mRNA assay | ARRDC3 | qSTAR qPCR Primer Pair | OriGene Technologies | HP213845; NM_020801 | Paired mRNA target for miR-182-5p; single melt-curve peak required |
| mRNA assay | FBLN5 | qSTAR qPCR Primer Pair | OriGene Technologies | HP209296; NM_006329 | Paired mRNA target for miR-200c-3p; single melt-curve peak required |
| mRNA assay | TACC1 | qSTAR qPCR Primer Pair | OriGene Technologies | HP230098; NM_006283 | Paired mRNA target for miR-342-3p; single melt-curve peak required |
| mRNA assay | RACGAP1 | qSTAR qPCR Primer Pair | OriGene Technologies | HP210444; NM_013277 | Paired mRNA target for miR-195-5p; single melt-curve peak required |
| mRNA assay | TPD52 | qSTAR qPCR Primer Pair | OriGene Technologies | HP202769; NM_001025252 | Paired mRNA target for miR-139-5p; single melt-curve peak required |
| mRNA assay | PARP1 | qSTAR qPCR Primer Pair | OriGene Technologies | HP205442; NM_001618 | Paired mRNA target for miR-125b-5p; single melt-curve peak required |
| mRNA assay | TPM3 | qSTAR qPCR Primer Pair | OriGene Technologies | HP226357; NM_152263 | Paired mRNA target for miR-145-5p; single melt-curve peak required |
| mRNA reference | GAPDH | qSTAR qPCR Primer Pair | OriGene Technologies | HP205798; NM_002046 | Used in geometric mean with ACTB for mRNA normalization |
| mRNA reference | ACTB | qSTAR qPCR Primer Pair | OriGene Technologies | HP204660; NM_001101 | Used in geometric mean with GAPDH for mRNA normalization |

Specimen-handling note: index breast-lesion tissue was obtained by core-needle biopsy or surgical excision before neoadjuvant systemic therapy. Preservation format was not uniformly encoded in the retained study database and was not used as an analytical variable.

**Supplementary Table 5.** Complete-case LASSO screening and marker-selection stability.

| **Marker** | **Selection frequency** | **Median coefficient** | **2.5th percentile** | **97.5th percentile** |
| --- | --- | --- | --- | --- |
| miR-205-5p | 1.00 | −0.533 | −0.865 | −0.286 |
| HMGB3 | 1.00 | 0.804 | 0.575 | 1.090 |
| miR-96-5p | 1.00 | 0.312 | 0.225 | 0.357 |
| FOXO1 | 1.00 | −0.424 | −0.622 | −0.253 |
| miR-21-5p | 1.00 | 0.420 | 0.311 | 0.539 |
| PIK3R1 | 1.00 | −0.963 | −1.245 | −0.577 |
| miR-182-5p | 1.00 | 0.526 | 0.271 | 0.834 |
| ARRDC3 | 0.00 | 0.000 | 0.000 | 0.000 |
| miR-200c-3p | 0.78 | 0.016 | 0.000 | 0.055 |
| FBLN5 | 0.52 | −0.059 | −0.300 | 0.000 |
| miR-342-3p | 1.00 | 0.416 | 0.169 | 0.740 |
| TACC1 | 1.00 | −0.462 | −0.814 | −0.205 |
| miR-195-5p | 1.00 | −0.565 | −0.770 | −0.328 |
| RACGAP1 | 0.00 | 0.000 | 0.000 | 0.000 |
| miR-139-5p | 0.00 | 0.000 | 0.000 | 0.000 |
| TPD52 | 0.52 | 0.069 | 0.000 | 0.368 |
| miR-125b-5p | 0.00 | 0.000 | 0.000 | 0.000 |
| PARP1 | 1.00 | 0.273 | 0.069 | 0.452 |
| miR-145-5p | 0.22 | 0.000 | −0.075 | 0.000 |
| TPM3 | 1.00 | 0.174 | 0.029 | 0.400 |

*The 20-marker complete-case screening set contained 77 of the 105 training records. No molecular imputation was used for this complete-case screen.*

**Supplementary Table 6.** Diagnosis-stratified train-validation repetitions.

| **Metric** | **Median** | **Q1** | **Q3** | **2.5th percentile** | **97.5th percentile** |
| --- | --- | --- | --- | --- | --- |
| Validation AUC | 0.918 | 0.890 | 0.936 | 0.848 | 0.974 |

The supplementary repeated-split AUC summary is analytically separate from the primary prespecified 100-split result reported in Supplementary Table 10. Split-specific outputs and repeated-fit coefficient summaries are retained in the companion analysis workbook.

**Supplementary Table 7.** Summary source values for revised Figure 4 and candidate-pair multiplicity correction.

| **miRNA–mRNA axis** | **Benign miRNA mean** | **Malignant miRNA mean** | **Benign mRNA mean** | **Malignant mRNA mean** | **Pearson r** | **Raw *P*** | **BH-adjusted *P*** |
| --- | --- | --- | --- | --- | --- | --- | --- |
| miR-205-5p/HMGB3 | 6.873 | 5.897 | 4.865 | 6.083 | −0.345 | <0.001 | <0.003 |
| miR-96-5p/FOXO1 | 5.342 | 6.170 | 6.775 | 5.864 | −0.354 | <0.001 | <0.003 |
| miR-21-5p/PIK3R1 | 6.171 | 6.914 | 6.466 | 5.742 | −0.185 | 0.023 | 0.029 |
| miR-182-5p/ARRDC3 | 5.475 | 6.221 | 6.184 | 5.680 | −0.497 | <0.001 | <0.003 |
| miR-200c-3p/FBLN5 | 6.079 | 6.740 | 6.391 | 5.813 | −0.206 | 0.014 | 0.023 |
| miR-342-3p/TACC1 | 5.844 | 6.566 | 5.497 | 4.968 | −0.257 | 0.002 | 0.004 |
| miR-195-5p/RACGAP1 | 6.055 | 5.426 | 5.587 | 5.792 | −0.188 | 0.026 | 0.029 |
| miR-139-5p/TPD52 | 5.606 | 5.057 | 5.883 | 6.590 | −0.199 | 0.017 | 0.024 |
| miR-125b-5p/PARP1 | 5.997 | 5.633 | 5.872 | 6.263 | −0.283 | <0.001 | <0.003 |
| miR-145-5p/TPM3 | 5.987 | 5.434 | 6.287 | 6.658 | −0.155 | 0.065 | 0.065 |

All expression values are normalized −ΔCt. Correlations are pooled descriptive statistics. For Benjamini–Hochberg adjustment, raw values reported as *P* < 0.001 were conservatively entered as 0.001; the corresponding adjusted values are therefore shown as upper bounds (<0.003). These pooled correlations should not be interpreted as within-group or covariate-adjusted evidence of direct regulation.

**Supplementary Table 8.** Five-marker training-set standardization parameters.

| **Marker** | **Training mean** | **Training SD** | **Coefficient** |
| --- | --- | --- | --- |
| miR-205-5p | 6.263 | 0.936 | −1.12 |
| HMGB3 | 5.595 | 1.207 | 1.03 |
| miR-96-5p | 5.752 | 1.064 | 0.88 |
| FOXO1 | 6.216 | 1.132 | −0.91 |
| miR-21-5p | 6.542 | 1.026 | 0.74 |

Intercept = 0.18; locked malignant-classification threshold = 0.52. Expression scale: normalized −ΔCt. The means and SDs shown here should be confirmed in the original training dataset before external score implementation.

**Supplementary Table 9.** Diagnostic classification metrics with 95% confidence intervals.

| **Cohort** | **Accuracy** | **Sensitivity** | **Specificity** | **PPV** | **NPV** | **Confusion counts (TN/FP/FN/TP)** |
| --- | --- | --- | --- | --- | --- | --- |
| Training | 0.829 (0.745–0.889) | 0.772 (0.648–0.862) | 0.896 (0.778–0.955) | 0.898 (0.782–0.956) | 0.768 (0.642–0.859) | 43/5/13/44 |
| Internal validation | 0.844 (0.712–0.923) | 0.840 (0.653–0.936) | 0.850 (0.640–0.948) | 0.875 (0.690–0.957) | 0.810 (0.600–0.923) | 17/3/4/21 |
| Pooled cohort, descriptive only | 0.833 (0.766–0.884) | 0.793 (0.693–0.866) | 0.882 (0.785–0.939) | 0.890 (0.798–0.943) | 0.779 (0.675–0.857) | 60/8/17/65 |

Confidence intervals for binomial classification measures were calculated using the Wilson method from the reported confusion-matrix counts.

**Supplementary Table 10.** Prespecified sensitivity analyses.

| **Analysis** | **Analytic Set** | **n** | **AUC** | **Change from Primary Internal-Validation AUC** | **Sensitivity** | **Specificity** | **Stability Interpretation** |
| --- | --- | --- | --- | --- | --- | --- | --- |
| Age + BI-RADS adjustment | Internal-validation cohort | 45 | 0.872 | −0.022 | 0.800 | 0.850 | Stable |
| Excluding ductal carcinoma in situ | Invasive malignant tumors and benign lesions only | 138 | 0.886 | −0.008 | 0.822 | 0.850 | Stable |
| RIN ≥6.0 and cellularity ≥40% | High-quality RNA subset | 122 | 0.902 | +0.008 | 0.846 | 0.864 | Stable |
| 100 repeated stratified splits | Repeated internal-validation estimates | 150 | 0.887, median; IQR 0.854–0.918 | −0.007 | 0.812 | 0.842 | Stable |

**Supplementary Table 11.** Within-group and covariate-adjusted correlations for the ten candidate axes.

| **Axis** | **Benign n** | **Benign r** | **Benign BH q** | **Malignant n** | **Malignant r** | **Malignant BH q** | **Pooled n** | **Partial r*** | **Partial BH q** |
| --- | --- | --- | --- | --- | --- | --- | --- | --- | --- |
| miR-205-5p/HMGB3 | 68 | −0.092 | 0.526 | 82 | −0.200 | 0.179 | 150 | −0.150 | 0.116 |
| miR-96-5p/FOXO1 | 68 | −0.095 | 0.526 | 82 | −0.345 | 0.008 | 150 | −0.231 | 0.017 |
| miR-21-5p/PIK3R1 | 68 | −0.142 | 0.411 | 82 | −0.026 | 0.816 | 150 | −0.085 | 0.306 |
| miR-182-5p/ARRDC3 | 64 | −0.492 | <0.001 | 80 | −0.441 | <0.001 | 144 | −0.460 | <0.001 |
| miR-200c-3p/FBLN5 | 65 | −0.252 | 0.107 | 79 | −0.041 | 0.803 | 144 | −0.142 | 0.134 |
| miR-342-3p/TACC1 | 66 | −0.202 | 0.209 | 78 | −0.149 | 0.276 | 144 | −0.159 | 0.116 |
| miR-195-5p/RACGAP1 | 66 | −0.324 | 0.040 | 78 | −0.064 | 0.726 | 144 | −0.175 | 0.096 |
| miR-139-5p/TPD52 | 66 | −0.090 | 0.526 | 78 | −0.161 | 0.276 | 144 | −0.125 | 0.174 |
| miR-125b-5p/PARP1 | 66 | −0.258 | 0.107 | 80 | −0.256 | 0.073 | 146 | −0.244 | 0.016 |
| miR-145-5p/TPM3 | 66 | −0.070 | 0.578 | 80 | −0.152 | 0.276 | 146 | −0.116 | 0.188 |

** Partial correlation controls for diagnosis, age, and target-lesion cellularity. Benjamini–Hochberg correction was applied within each family of ten tests.*

**Supplementary Table 12.** Comparison of BI-RADS, clinical, molecular, and combined models in the validation partition.

| **Model** | **AUC** | **Bootstrap 95% CI** | **Brier** | **Calibration intercept** | **Calibration slope** | **DCA range** |
| --- | --- | --- | --- | --- | --- | --- |
| BI-RADS | 0.835 | 0.721–0.923 | 0.147 | 0.119 | 1.221 | 0.10–0.90 |
| Clinical | 0.914 | 0.808–0.985 | 0.119 | 0.265 | 0.986 | 0.15–0.90 |
| Molecular | 0.900 | 0.800–0.975 | 0.132 | 0.800 | 0.750 | 0.20–0.90 |
| Combined | 0.966 | 0.915–1.000 | 0.086 | 0.805 | 1.097 | 0.10–0.90 |

Clinical model: age + menopausal status + lesion size + BI-RADS. Molecular model: five-marker score. Combined model: clinical variables + molecular score. These values are exploratory supplementary comparisons and do not replace the primary internal-validation AUC reported in the main manuscript.

**Supplementary Table 13.** Exploratory BI-RADS 4 subgroup performance.

| **Set** | **n** | **Benign** | **Malignant** | **AUC** | **Bootstrap 95% CI** | **TN** | **FP** | **FN** | **TP** |
| --- | --- | --- | --- | --- | --- | --- | --- | --- | --- |
| All BI-RADS 4 | 70 | 28 | 42 | 0.921 | 0.849–0.974 | 25 | 3 | 8 | 34 |
| Validation BI-RADS 4 | 23 | 9 | 14 | 0.905 | 0.762–1.000 | 8 | 1 | 3 | 11 |

**Supplementary Table 14.** Bootstrap calibration uncertainty for the molecular score.

| **Metric** | **Estimate** | **Bootstrap 95% CI** |
| --- | --- | --- |
| Calibration intercept | 0.800 | −0.319 to 1.875 |
| Calibration slope | 0.750 | 0.481–1.527 |
| Brier score | 0.132 | 0.066–0.207 |

**Supplementary Table 15.** Public-source TCGA correlation context for the ten measured axes.

| **Axis** | **TCGA Pearson r** | ***P* value** |
| --- | --- | --- |
| miR-21-5p/PIK3R1 | −0.170 | 8.02×10−9 |
| miR-182-5p/ARRDC3 | −0.164 | 2.70×10−8 |
| miR-96-5p/FOXO1 | −0.269 | 2.33×10−20 |
| miR-200c-3p/FBLN5 | −0.123 | 3.26×10−5 |
| miR-342-3p/TACC1 | −0.130 | 1.02×10−5 |
| miR-195-5p/RACGAP1 | −0.256 | 1.46×10−18 |
| miR-139-5p/TPD52 | −0.229 | 4.58×10−15 |
| miR-205-5p/HMGB3 | −0.093 | 1.58×10−3 |
| miR-125b-5p/PARP1 | −0.237 | 4.83×10−16 |
| miR-145-5p/TPM3 | −0.209 | 8.87×10−13 |
